# Genus *Indiopius* Fischer, 1966 (Hymenoptera, Braconidae, Opiinae) in Iran with a key to the world species

**DOI:** 10.3897/zookeys.368.6658

**Published:** 2014-01-08

**Authors:** Francisco Javier Peris-Felipo, Zahra Rahmani, Sergey A. Belokobylskij, Ehsan Rakhshani

**Affiliations:** 1 Laboratory of Entomology and Pest Control, Institute Cavanilles of Biodiversity and Evolutionary Biology, University of Valencia, c/.Catedrático José Beltrán n 2, 46980 Paterna, Valencia, Spain; 2 Department of Plant Protection, College of Agriculture, University of Zabol, Zabol, P.O. Box: 98615-538, I. R. Iran; 3 Zoological Institute Russian Academy of Sciences, St. Petersburg 199034, Russia; 4 Museum and Institute of Zoology Polish Academy of Sciences, Wilcza 64, Warszawa 00-679, Poland

**Keywords:** Braconidae, Opiinae, *Indiopius*, new records, key, Iran

## Abstract

The Iranian species belonging to the genus *Indiopius* Fischer are reviewed. A description of the first recorded female of *I. cretensis* Fischer, 1966 is provided. A key to the world species of the genus *Indiopius* is given.

## Introduction

The subfamily Opiinae contains approximately 2,000 catalogued species worldwide (Yu et al. 2012). These are strictly koinobiont parasitoids of the Diptera-Cyclorrhapha (Wharton 1999), mainly of larvae of leaf miners and those living in fruits. The hosts are known for only about 300 opiine species, mostly belonging to the dipterous families Agromyzidae, Anthomyiidae and Tephritidae (Fischer 1971, 1972, 1977, 1987; Shaw and Huddleston 1991).

The genus *Indiopius* Fischer, 1966 is a small and rarely collected taxon, with only eight known species, despite its wide distribution throughout the Afrotropical, Oriental and Palaearctic regions (Yu et al. 2012). The main characters for diagnosis of this genus are: marginal cell of the fore wing widely open apically; veins m-cu, r-m and 2-SR of the fore wing absent; the first subdiscal cell of the fore wing open postero-apically; vein cu-a of the hind wing absent; clypeus wide, short and impressed ventrally; mandible long and slender; occipital carina completely absent; the first to third metasomal tergites more or less distinctly coriaceous or rugulose; fourth to six metasomal segments usually largely retracted (Li et al. 2013).

Our investigation of the braconid parasitoid wasps of the subfamily Opiinae in Iran allowed the discovery of the genus *Indiopius* Fischer; one species is described from Iran for the first time. The description of the female of *Indiopius cretensis*, only the male was known until now, and a key for identification of the world species of *Indiopius* are included in this paper.

## Material and methods

Specimens were collected using standard sweeping nets on semi-aquatic plants within a protected landscape in the Sistan area (31°02'N, 61°32'E, 485 m A.S.L). This small area is artificially irrigated to protect the endemic flora and fauna from the unfavorable dry climates of the recent decade.

A field emission gun environmental scanning electron microscope (Hitachi S-4100) at 2 kV was used for high-resolution imaging without gold coating.

For the terminology of the morphological features and sculpture, measurements and wing venation nomenclature, see van Achterberg (1988, 1993). Additionally, the following abbreviations are used: POL – postocellar line; OOL – ocular-ocellar line; and OD – maximum diameter of lateral ocellus. The specimens studied are deposited in the collections of the Faculty of Agriculture, University of Zabol, Iran (FAOUZ), in the Entomological Collection at the University of Valencia (Valencia, Spain; ENV), and in the Zoological Institute RAS (St. Petersburg, Russia; ZISP).

## Taxonomy

### Indiopius cretensis

Fischer, 1983

http://species-id.net/wiki/Indiopius_cretensis

Figures 1 – 15

Indiopius cretensis Fischer 1983: 1; Yu et al. 2012.

#### Material examined.

1 ♂ (holotype), Greece, Crete, Biro, Canea, 1906/11 (Hungarian Natural History Museum, Budapest); 2 ♀ and 4 ♂, Iran, Zabol (31°02'28"N, 61°32'02"E, 482 m A.S.L.), 26.iv.2013, sweeping on *Cyperus rotundus* (Z. Rahmani leg.) (ENV, ZISP); 7 ♀ and 20 ♂, same locality, 22–24, 26 and 27.iv.2013 (Z. Rahmani leg.) (FAUOZ).

#### Diagnosis.

This species resembles *Indiopius fischeri* Samiuddin et Ahmad from India and *Indiopius turcmenicus* Tobias from Turkmenistan. *Indiopius cretensis* differs from *Indiopius fischeri* in has the maxillary palpi as long as head height (0.5 times in *Indiopius fischeri*), the first flagellar segment of female 2.55–2.65 times as long as its width (2.1 times in *Indiopius fischeri*), and the middle flagellar segments of female 2.25–2.65 times as long as their width (1.5 times in *Indiopius fischeri*). Also, *Indiopius cretensis* differs from *Indiopius turcmenicus* in having the first flagellar segment of female 2.55–2.65 times as long as its width (3.0 times in *Indiopius turcmenicus*), the middle flagellar segments of female 2.25–2.65 times as long as their width (2.0 times in *Indiopius turcmenicus*), the first metasomal tergite 1.0–1.1 times as long as its apical width (0.8 times in *Indiopius turcmenicus*), and vein 1cu-a postfurcal (interstitial in *Indiopius turcmenicus*).

#### Description.

Female (first record). Body length 1.0–1.1 mm; fore wing length 1.4–1.5 mm.

*Head*. In dorsal view, 2.0 times as wide as median length, 1.4 times as wide as mesoscutum, smooth, with rounded temples behind eyes. Eye in lateral view 1.35 times as high as wide and twice as wide as temple in middle. POL 2.6 times OD; OOL 3.45 times OD. Face 1.25 times as wide as high; inner margins of eyes subparallel. Clypeus 3.35 times as wide as high, slightly curved ventrally. Mandible narrow, weakly and evenly widened towards base; upper tooth longer than lower tooth. Maxillary palpi as long as head height. Antenna thick, 18-segmented. Scape 1.40–1.45 times as long as maximum width, about twice as long as pedicel. First flagellar segment 2.55–2.65 times as long as its apical width, 1.15–1.20 times as long as second segment; second segment 2.6–2.7 times as long as its maximum width. Third to ninth flagellar segments 2.55–2.65 times and tenth to sixteenth segments 2.25–2.30 times as long as their maximum width.

*Mesosoma*. In lateral view, 1.05 times as long as high. Mesoscutum 0.75–0.80 times as long as its maximum width. Notauli mainly absent, finely developed on vertical anterior part. Mesoscutal pit absent. Prescutellar depression with numerous carinae. Precoxal suture present, very shallow, not reaching anterior and posterior margins of mesopleuron. Posterior mesopleural furrow smooth. Propodeum completely smooth. Propodeal spiracles relatively small.

*Legs*. Hind femur 3.60–3.65 times as long as its maximum width. Hind tibia weakly widened towards apex, about 10.0 times as long as its maximum subapical width, 1.1 times as long as hind tarsus. First segment of hind tarsus 1.3 times as long as second segment.

*Wings*. Length of fore wing 2.5 times its maximum width. Pterostigma almost triangular. Vein 1-R1 not reaching wing apex and as long as pterostigma. Veins r, 3-SR and SR1 not differentiated; 1-M straight; cu-a postfurcal, 1-CU1 widened. First subdiscal cell open. CU1b absent. M+CU1 only apically sclerotized. Hind wing 6.5 times as long as its maximum width; vein cu-a absent.

*Metasoma*. Distinctly depressed dorso-ventrally, apical segments rather distinctly protruding behind third tergite. First tergite weakly widened towards apex, 1.1 times as long as its apical width, finely rugose but basally smooth. Second tergite largely finely granulate. Third and following tergites smooth. Ovipositor 1.05 times as long as first tergite, 0.65 times as long as hind femur.

*Colour*. Body and legs uniformly brown to dark brown, second tergite yellowish brown. Wings hyaline. Pterostigma brown.

*Male*. Body length 1.4–1.5 mm; fore wing length 1.5 mm. First flagellar segment 2.7–2.8 times and second segment 2.5 times as long as their width accordingly. Third to sixteenth flagellar segments 2.20–2.75 times as long as their width. Otherwise similar to female.

**Figures 1–6.**
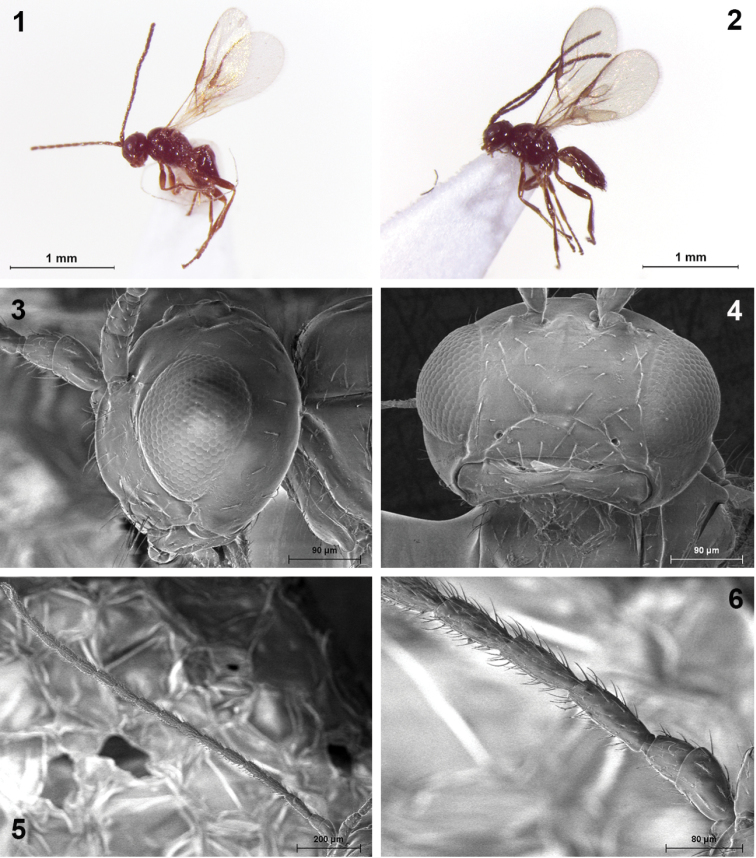
*Indiopius cretensis* Fischer (**1, 3–6** female **2** male). **1, 2** Habitus, lateral view **3** Head, lateral view **4** Face in front view, mandible and maxillary palpi **5** Antenna **6** Basal segments of antenna.

**Figures 7–12.**
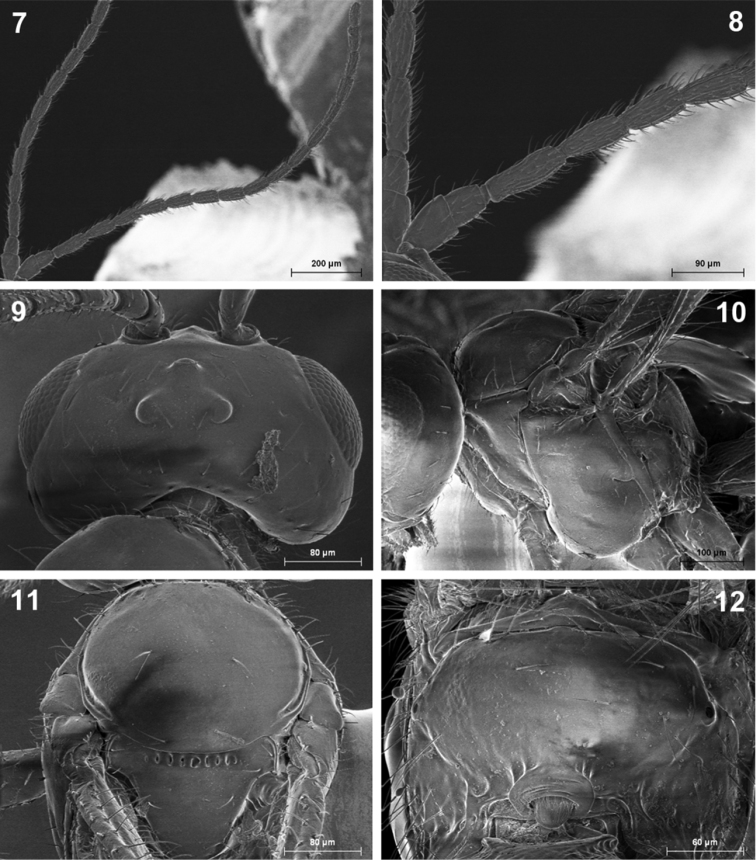
*Indiopius cretensis* Fischer (**7, 8** male **9–12** female). **7** Antenna **8** Basal segments of antenna **9** Head, dorsal view **10** Mesosoma, lateral view **11** Mesoscutum **12** Propodeum.

**Figures 13–15.**
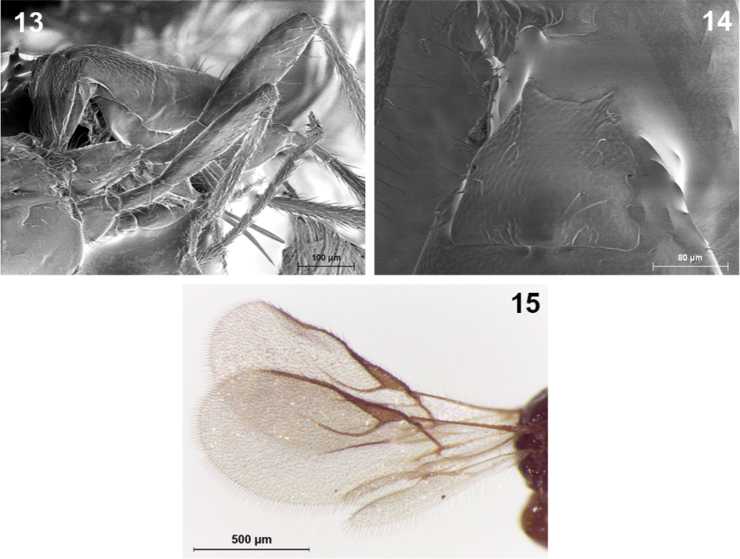
*Indiopius cretensis* Fischer (female). **13** Hind and middle legs, metasoma and ovipositor, lateral view **14** First metasomal tergite **15** Fore and hind wings.

#### Distribution.

Cape Verde Islands, Greece, Turkey, and Iran (new record).

### Key to the world species of the genus *Indiopius* Fischer

**Table:** 

1	Precoxal suture long, reaching anterior or anterior and posterior margins of mesopleuron	2
–	Precoxal suture short, not reaching anterior and posterior margins of mesopleuron	3
2(1)	Maxillary palpi 0.6 times as long as head height. Hind femur 3.3 times as long as its maximum width. First flagellar segment 2.2 times as long as its maximum width. Head in dorsal view 1.75 times as wide as median length. Antenna 19–segmented. Body length 1.3 mm. China	*Indiopius chenae* van Achterberg & Li (♀)
–	Maxillary palpi as long as head height. Hind femur 4.0 times as long as its maximum width. First flagellar segment 2.5 times as long as its maximum width. Head in dorsal view 2.0 times as wide as median length. Antenna 19–segmented. Body length 1.4 mm. Turkey, Vietnam	*Indiopius saigonensis* Fischer (♀)
3(1)	Hind femur wide, 3.60–3.65 times as long as its maximum width	4
–	Hind femur narrow, 4.0–4.5 times as long as its maximum width	6
4(3)	First flagellar segment 2.1 times as long as its maximum width. Middle flagellar segments 1.5 times as long as their width. Maxillary palpi 0.5 times as long as head height. – Vein 1 cu-a clearly postfurcal. Antennae 18–segmented. Body length 1.3 mm. India	*Indiopius fischeri* Samiuddin & Ahmad (♀♂)
–	First flagellar segment 2.65–3.00 times as long as its maximum width. Middle flagellar segments 2.00–2.55 times as long as their width. Maxillary palpi as long as head height	5
5(4)	First flagellar segment 2.65 times as long as its maximum width. Middle flagellar segments 2.25–2.55 times as long as their width. First metasomal tergite 1.0–1.1 times as long as its apical width. Vein 1 cu-a postfurcal. Antennae 18–20–segmented. Body length 1.0–1.5 mm. Cape Verde Islands, Greece, Turkey, Iran	*Indiopius cretensis* Fischer (♀♂)
–	First flagellar segment 3.0 times as long as its maximum width. Middle flagellar segments about 2.0 times as long as their width. First metasomal tergite 0.8 times as long as its apical width. Vein 1 cu-a interstitial. Antennae more than 14–segmented (missing apically). Body length 1.3 mm. Turkmenistan	*Indiopius turcmenicus* Tobias (♀♂)
6(3)	First flagellar segment 2.5 times as long as its width. Middle flagellar segments about 1.5 times as long as their width. – Maxillary palpi as long as head height. Hind femur 4.5 times as long as its maximum width. Vein 1 cu-a almost interstitial. Antennae 19–segmented. Body length 1.7 mm. India	*Indiopius humillimus* Fischer (♀)
–	First flagellar segment 3.0 times as long as its width. Middle flagellar segments about twice as long as their width	7
7(6)	Maxillary palpi 0.7 times as long as head height. First metasomal tergite 1.2 times as long as its apical width. Head in dorsal view 1.7 times as wide as its median length. Antenna 18–19–segmented. Body length 1.3–1.4 mm. China	*Indiopius alucitacius* Weng & Chen (♀)
–	Maxillary palpi as long as head height. First metasomal tergite as long as its apical width. Head in dorsal view 1.9 times as wide as its median length. Antenna 18–segmented. Body length 1.1 mm. Turkey	*Indiopius yilmazae* Fischer & Beyarslan (♀)

## Acknowledgments

The contribution of Ehsan Rakhshani was partially supported by the grant No. 89–9198, University of Zabol, and of Sergey Belokobylskij by grant of the Russian Foundation for Basic Research (No. 13–04–00026).

## Supplementary Material

XML Treatment for Indiopius cretensis
